# A Novel Method and an Equipment for Generating the Standard Moisture in Gas Flowing through a Pipe

**DOI:** 10.3390/s18103438

**Published:** 2018-10-13

**Authors:** Yusuke Tsukahara, Osamu Hirayama, Nobuo Takeda, Toru Oizumi, Hideyuki Fukushi, Nagisa Sato, Toshihiro Tsuji, Kazushi Yamanaka, Shingo Akao

**Affiliations:** Ball Wave Inc., T-Biz 503, 6-6-40, Aza Aoba, Aramaki, Aoba, Sendai, Miyagi 980-8579, Japan; oflathill@gmail.com (O.H.); takeda@ballwave.jp (N.T.); oizumi@ballwave.jp (T.O.); fukushi@ballwave.jp (H.F.); n.is2v.w@gmail.com (N.S.); t-tsuji@material.tohoku.ac.jp (T.T.); yamanaka@ballwave.jp (K.Y.); akao@ballwave.jp (S.A.)

**Keywords:** Trace moisture, ball SAW sensor, surface acoustic wave, permeation tube

## Abstract

When inert gas containing water molecules flows into a metal pipe, the water molecules cannot exit instantaneously from the outlet of the pipe but are captured at adsorption sites on the inner surface of the pipe until most of the sites are occupied. A theoretical model and a subsequent experiment in this article show that the delay time depends on the amount of moisture level; the higher the moisture-level, the shorter the delay time. Based on the result, we propose a new method and its implementation to the validation of a standard moisture generation to be used in the field measurement such as in factories and pipe lines.

## 1. Introduction

The measurement and control of trace moisture in gaseous materials are an important step for the quality enhancement in manufacturing semiconductors and light emitting displays [1]. There are various technologies for trace moisture measurement including aluminum oxide sensors [2], tunable laser diodes [3], and the cavity ring down spectroscopy [4]. Recently, the present authors developed a new technology called ball surface acoustic wave (SAW) moisture sensors [5]. It covers a wide range of moisture level, from a few ppbV to hundreds ppmV, and the most prominent characteristic is its quick response to a sudden variation in the moisture level [6].

For any methods of moisture measurement, calibration is inevitable, and the calibration of a particular sensor should be traceable to an international standard [7]. It is also important to periodically validate the sensor accuracy against the calibrated values while the sensor is running in the field of measurement such as in factories and in pipe lines [8]. To calibrate and to validate the moisture sensors, the accurate generation of moisture at certain predetermined values is crucial. Different methods have been proposed and implemented including the diffusion tube method [9], NPL method [10] and a method using permeation tubes [11].

Of these methods, the one which uses the permeation tube is suitable to be implemented in an equipment for the on-site validation because of its small volume. The permeation tube is made of a polymer tube with a certain diameter and a length containing liquid water in it. A tightly sealed cell containing the permeation tube is connected to a pipe line, and the gas flows into and out of the cell at a regulated constant flow rate. The polymeric material is permeable to water molecules, and it therefore dispenses the water molecules at a constant rate when the temperature and pressure are maintained constant. The amount of moisture generated by the permeation tube is controlled by changing the temperature. As a result, the amount of moisture in the output gas from the cell is a sum of the original moisture in the input gas and that generated by the permeation tube. Therefore, the output gas can be used as a standard moisture for the validation when we can guarantee that the amount of moisture contained in the input gas is small enough. To this end, dryers containing desiccants such as silica particles are used. However, the dryer stops absorbing the water molecules when it becomes saturated, therefore we need to know if the dryer is functioning properly.

In this paper, we propose a new method and its implementation to guarantee that a dryer connected to the inlet of the cell containing the permeation tube is working properly. By using the new method, the validation of the dryer output gas using the ball SAW moisture sensor is easily achieved in the field measurement such as in factories and pipe lines.

## 2. Moisture in a Gas Flowing through a Pipe

In the first place, we analyze the behavior of water molecules in a gas flowing through a metal pipe. The water molecules are readily adsorbed to the inner surface of pipes, cylinders, and chambers when a gas containing moisture flows through them. The water molecules are one of the contaminants seriously affecting the quality of products processed using these pipes, cylinders, and chambers [12]. It has been shown that a smaller amount of water molecule is adsorbed on a smoother inner surface of a metal pipe [13]. Recently, the present authors showed [14] that a quantitative analysis of correlation was possible between the degree of surface treatment such as electrochemical buffing (ECB) and electropolishing (EP), and the amount of water adsorption when a ball SAW moisture sensor monitored the time-dependence of moisture in a gas passing through a metal pipe only 10 cm long. This was made possible because the ball SAW sensor had a quick response time within a few seconds. In the following, we propose a theoretical model to describe the time-dependence of the moisture level in an infinitesimally small volume of an inert gas that is flowing through a pipe.

## 3. Theoretical Analysis

There have been theoretical and experimental studies on the behavior of molecules contained in a carrier gas passing through a column in a gas chromatograph [15,16]. However, the strength of the interaction of those molecules with the inner surface of the column is basically a linear function of the number of the molecules, and the secondary nonlinear effect was taken into account for the detail analysis of deviation from the linear model. This correctly reflected the most prominent feature of gas chromatography that the retention time is independent of the number of molecules of interest. In contrast, the adsorption to, and desorption from, the metal surface of water molecules seem to be fundamentally nonlinear function of the moisture, as shown in the following. A detail of the model is found in the Appendix A.

**Figure 1:**
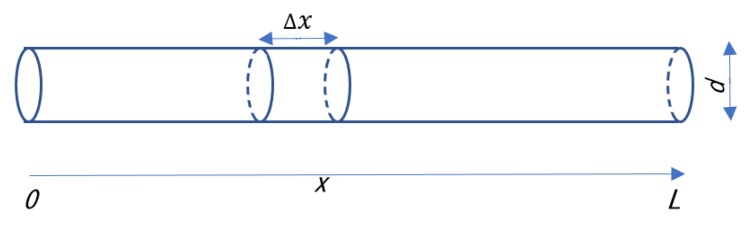
Inert gas containing moisture flows through a pipe with a length L and an inner diameter d.

Let us assume that an inert gas is flowing at constant flow rate f (m^3^ s^−1^) through a pipe with a length L (m) and an inner diameter d (m) as depicted in Figure 1. The surface density of adsorption sites is s (mol m^−2^), and an adsorption ratio to the sites, or the ratio of the number of adsorption sites occupied by the water molecules to the total number of adsorption sites, is r. The normalized moisture in the gas is W, that is

(1) W= (w×L^3)/(s×L^2 )

where w is the moisture measured in $\left({\mathrm{mol}\;m}^{-3}\right)$. A set of two normalized dimensionless equations is given as follows where a and b are the only adjustable parameters, as shown in the Appendix A (Equations (A9) and (A10)).

(2) $$\frac{\partial r}{\partial \tau }=-a\times r+b\times \left(1-r\right)\times W\;\frac{\partial W}{\partial \tau }+\frac{\partial W}{\partial \xi }=g\times a\times r-g\times b\times \left(1-r\right)\times W$$

where τ and ξ are normalized time and space coordinate, respectively, defined by Equations (A11) and (A12) in the Appendix A.

(3) $$\tau =\;\frac{4\times t\times f}{\pi \times L\times d^{2}}$$

(4) $$\xi =\;\frac{x}{L}$$

where t and x are time and space coordinates measured in (s) and (m), respectively. A computer program was developed in Fortran language to numerically solve the equations.

To simulate the experiments in [14], we set the values of parameters as follows.

f: 0.1 L/min

L: 10 cm

d: 4.35 mm

w_0_: 1 ppbV

w_1_: 1 ppmV

**Figure 2:**
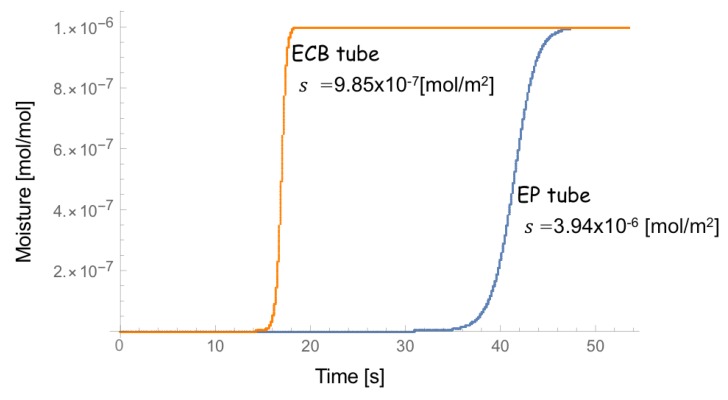
Calculated time-dependence of moisture at the outlet of the pipe for electrochemical buffing (ECB) tube (orange) and electropolishimg (EP) tube (blue), respectively.

Assuming a = 1 and b = 1 (for simplicity) and adjusting s, we obtain the time-dependence of moisture measured at the outlet of the pipe for ECB and EP tubes, respectively, as shown in Figure 2. The leading edges of time-dependence of moisture for ECB and EP tubes reasonably match the experimental values of 15 (s) and 40 (s), respectively, in Reference [14].

**Figure 3:**
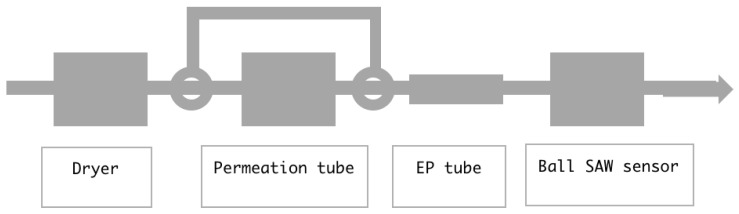
Inert gas with unknown moisture passes through a dryer, then flows into a cell containing a permeation tube and then into a cell for the ball surface acoustic wave (SAW) moisture sensor.

Now we simulate the behavior of moisture in a setting depicted in Figure 3, where an inert gas with unknown moisture passes through a dryer and then flows into a cell containing a permeation tube. The gas coming out of the cell goes into an EP tube with L = 16 (cm). We chose the EP tube because it would cause a long delay time, which was easy to detect. We assume that the temperature of the permeation tube is controlled so that it generates 1 or 5 ppmV of moisture. Then, we solve the equations for the moisture in the EP tube with a set of different initial conditions which simulates the uncontrollable variation of the moisture coming out of the dryer.

**Figure 4:**
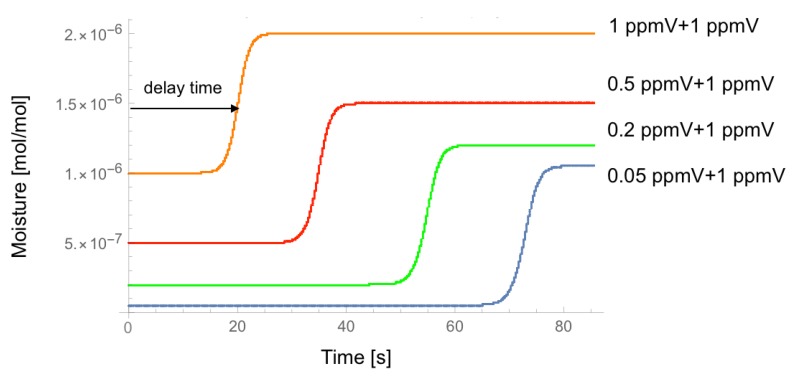
Calculated time-dependence of moisture at the outlet of the EP tube for the different set of initial moisture conditions at the inlet of the EP tube.

Figure 4 shows the calculated time-dependence of moisture at the outlet of the EP tube for the different set of initial conditions at the inlet of the EP tube. This simulates the situation where the output of the dryer contains the moisture of 0.05, 0.20, 0.50, and 1.00 ppmV, respectively, and then the permeation tube adds 1 ppmV of moisture. In Figure 4, we can see that the dryer’s performance can be evaluated by measuring the delay time between the onset of gas flow and the leading edge of the moisture change at the outlet of the EP tube.

**Figure 5:**
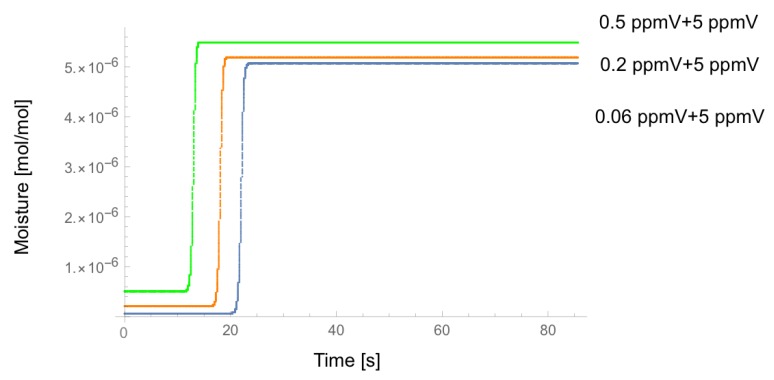
Calculated time-dependence of moisture at the outlet of the EP tube where the output of the dryer contains the moisture of 0.06, 0.20 and 0.50 ppmV, respectively, and then the permeation tube adds 5 ppmV of moisture.

Figure 5 shows the similar analysis where the output of the dryer contains the moisture of 0.06 ppmV, 0.2 ppmV, and 0.5 ppmV, respectively, and then the permeation tube adds 5 ppmV moisture. It is still valid that the dryer’s performance can be evaluated by measuring the delay time between the onset of gas flow and the leading edge of the moisture change at the outlet of the EP tube, though the time difference is smaller for the larger moisture.

## 4. Result

**Figure 6:**
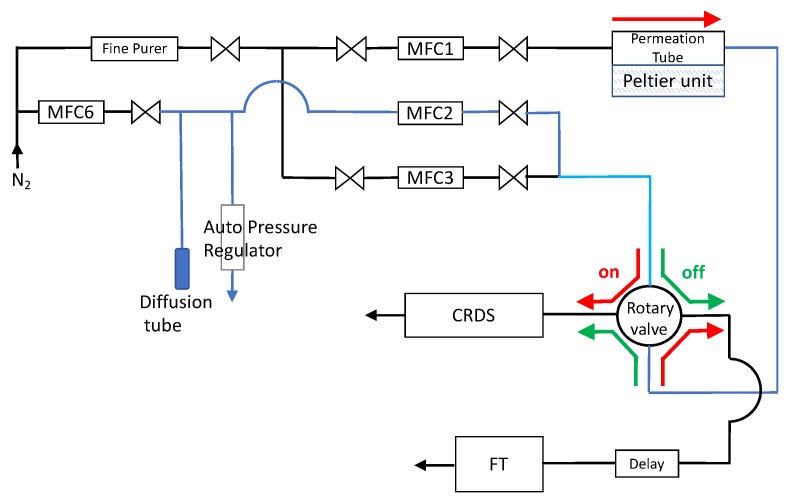
Experimental setup. MFC’s: mass flow controllers, Fine Pure: a dryer, CRDS: a CRDS Trace Gas Analyzer, Delay: a 10 (cm) long EP tube, FT: a ball SAW moisture sensor.

Figure 6 shows the experimental setup to validate the theoretical prediction. Nitrogen gas with controlled values of moisture is fed into a cell containing a permeation tube, and then goes into a 10 (cm) long EP tube, depicted as Delay. The gas coming out of the EP tube flows through a metal-mesh filter for removal of particles before reaching the measurement cell of ball SAW moisture sensor, depicted as FT. MFC1~MFC3 and MFC6 are mass flow controllers. Fine Purer is a dryer marketed by Osaka Gas Liquid Co., Ltd. in Osaka, Japan. Its specification declares that the gas coming out of it contains “< 1 nmol/mol for H_2_O”, which is less than 1 ppbV. Diffusion tube is providing the water molecules into the piping system. The “CRDS” block represents a CRDS Trace Gas Analyzer, HALO 3 H_2_O by Tiger Optics in Pennsylvania, USA, used as a reference. Its specification declares that the detection range and the low detection limit for H_2_O in nitrogen are 0–20 ppmV and 0.6 ppbV, respectively. In the numerical calculation in the previous section, the effect of the metal-mesh filter was taken into account by assuming the 16 (cm) long EP tube. The ball SAW sensor was driven with an electric pulse containing two different frequency components, namely 80 and 240 MHz. The two frequency components of the output signal were subtracted to compensate for the temperature dependence of the sensor, and then converted to the values of moisture content.

**Figure 7:**
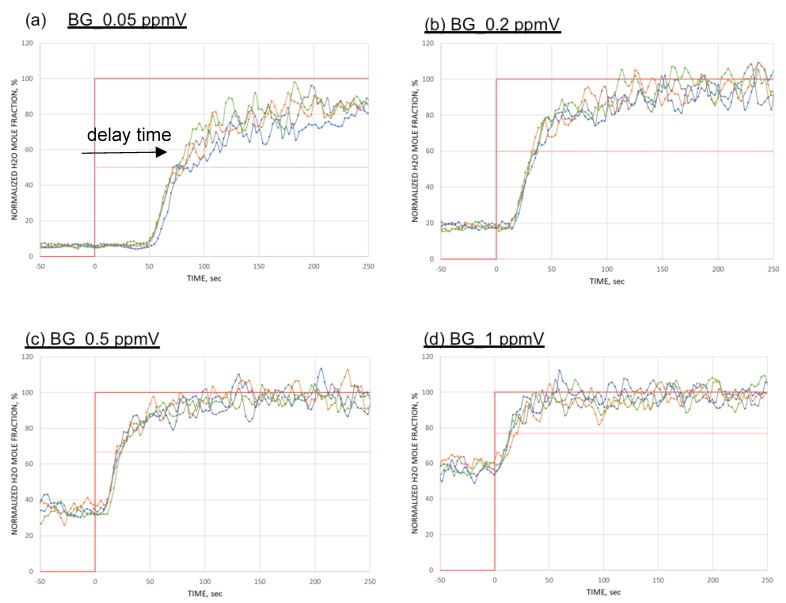
Measured signals of ball SAW moisture sensor for four different conditions: (**a**) 0.05, (**b**) 0.2, (**c**) 0.5, and (**d**) 1.0 ppmV of background moisture, each time mixed with 1 ppmV from permeation tube. The vertical axis is for normalized values. BG stands for back ground moisture.

Figure 7 shows the measured signals of the ball SAW moisture sensor for four different conditions: (a) 0.05, (b) 0.2, (c) 0.5, and (d) 1.0 ppmV of background moisture, each time mixed with 1 ppmV from permeation tube. The vertical axis is the normalized value of moisture measured by the ball SAW sensor because the absolute value is not calibrated yet. The experiment was repeated for four times with each condition.

**Figure 8:**
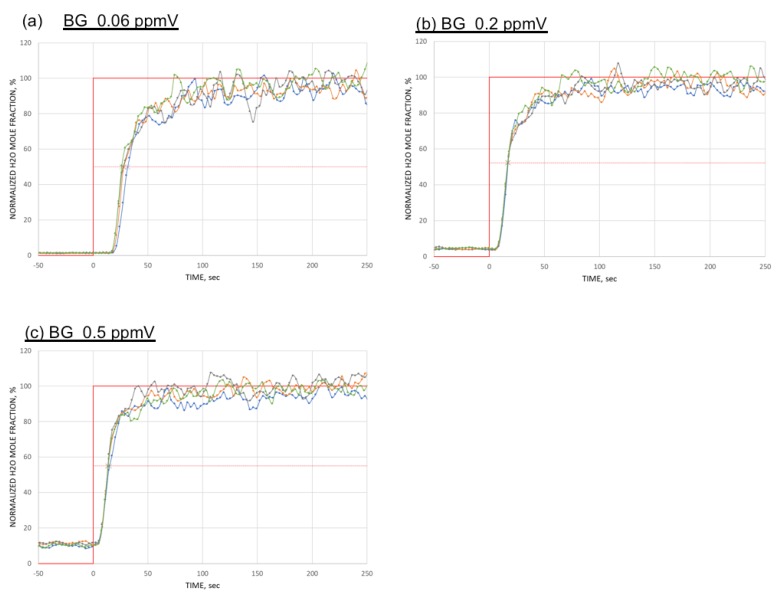
Measured signals for (**a**) 0.06, (**b**) 0.2, and (**c**) 0.5 ppmV of background moisture, each time mixed with 5 ppmV from permeation tube. The vertical axis is for normalized values. BG stands for back ground moisture.

Figure 8 show the measured signals for three different conditions: (a) 0.06, (b) 0.2, and (c) 0.5 ppmV of background moisture, each time mixed with 5 ppmV from permeation tube. It should be noted that the delay time between the onset and the leading edge of the moisture change depends on the background moisture as predicted by the theoretical simulation.

**Figure 9:**
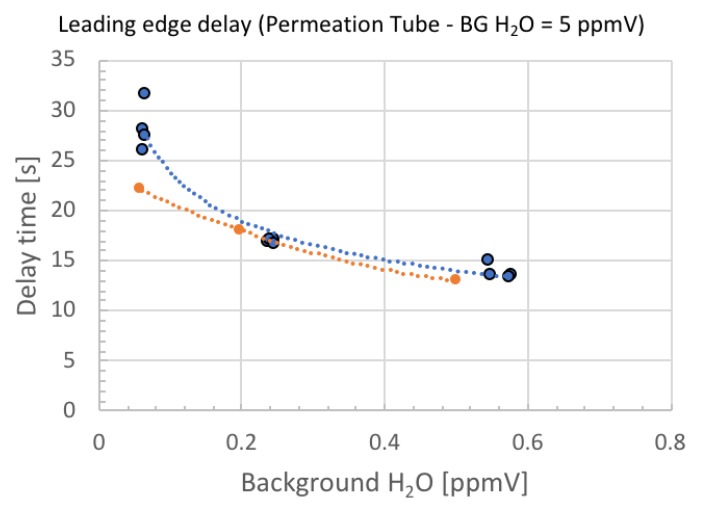
Theoretical (orange) and experimental (blue) delay time plotted as a function of background moisture when the permeation tube generates 5 ppmV moisture. Dotted lines are fitted curves.

**Figure 10:**
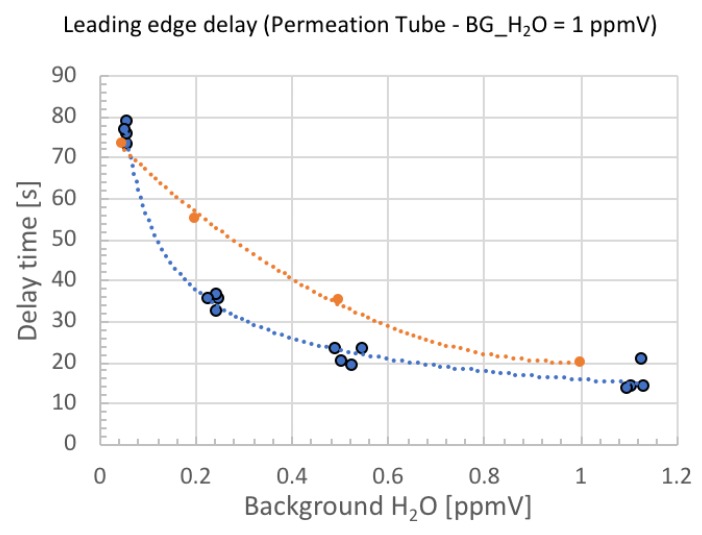
Theoretical (orange) and experimental (blue) delay time plotted as a function of background moisture when the permeation tube generates 1 ppmV moisture. Dotted lines are fitted curves.

More quantitatively, the theoretical and experimental delay time is plotted in Figure 9 and Figure 10. The theoretical and experimental values do not exactly match, but the trend is reproduced correctly and the smaller the background moisture, the larger the delay time.

## 5. Discussion

In this article, it is established that choice of a metal pipe with proper inner surface treatment in combination with a ball SAW moisture sensor can be used for the evaluation of a background moisture in a gas coming out of a dryer. This is an original novel design of a standard moisture generator with a permeation tube, particularly suitable for the validation of trace moisture sensors in the field measurement, such as in factories and in pipelines.

The experimental data in Figure 9 shows that the background moisture of 0.5 ppmV added to the 5 ppmV standard moisture gave rise to the time delay of −50%. In Figure 10, the background moisture of 0.1 ppmV added to the 1 ppmV standard moisture gave rise to the time delay of −27%. Therefore, we conclude that by measuring the delay time, we can easily distinguish the uncontrollable background moisture at less than 10%. This is a unique way of guaranteeing the accuracy of the standard moisture for the validation of ball SAW moisture sensors.

## Appendix A

Let us assume that an ideal gas containing water molecules flows through a pipe with a length L [m] and an inner diameter d [m] at a constant flow rate $f\;\left[m^{3}{\;s}^{-1}\right]$. We assume that the flow velocity is uniform over the entire cross-section of the pipe for simplicity. Then, the flow velocity v is

(A1) $$v=\frac{4\times f}{\pi \times d^{2}}$$

We propose that there are microscopic sites on the inner surface of the metal pipe with a surface density $s\;\left[{\mathrm{mol}\;m}^{-2}\right]$ where water molecules can be adsorbed. The water molecules attach to and detach from these sites at each instant, and on average there are s×r sites with water molecules adsorbed per unit area, where r is an adsorption ratio.

In the experiment [14], the nitrogen gas with a constant moisture $w_{0}\;\left({\mathrm{mol}\;m}^{-3}\right)$ flows into the pipe from the inlet (x=0). The moisture at the outlet (x=L) eventually becomes equal to that at the inlet after a sufficiently long time. Then, the moisture of the gas flowing into the inlet is suddenly changed to another constant value $w_{1}\;\left({\mathrm{mol}\;m}^{-3}\right)$ at time t=0, and the time dependence of the moisture at the outlet (w(t, x=L)) is monitored.

The amount of water molecules that detach from the unit area of the inner surface and enter into the carrier gas is proportional to the amount of adsorbed water molecules on the unit area, s×r. Thus, introducing a desorption coefficient $k_{d}$, it is $s\times r\times k_{d}$. On the other hand, the amount of water molecules adsorbed on the unit area of the inner surface is proportional to the product of the number of vacant sites, s×(1−r), and the number of water molecules in the carrier gas or moisture, w. Thus, it is $s\times \left(1-r\right)\times k_{a}\times w$ where $k_{a}$ is an adsorption coefficient.

Now let us take a volume with a length Δx at a position $x=x_{0}$ along the length of the pipe. The amount of water molecules contained in the volume increases due to the incoming flow from the upstream and decreases due to the outgoing flow to the downstream, and the net increase due to the flow during a time interval Δt is their difference,

(A2) $$-\frac{\partial w\left(t,\;x\right)}{\partial x}\times v\times {\left(\frac{d}{2}\right)}^{2}\times \pi \times \Delta x\times \Delta t$$

In this time interval, a part of the water molecules in the gas flow are adsorbed on the metal surface and a part of the water molecules on the surface detach from the surface, and the net amount of adsorbed molecules is,

(A3) $$\left[s\;k_{d}\;r\left(t,\;x\right)-s\;k_{a}\;\left(1-r\left(t,\;x\right)\right)\;w\left(t,\;x\right)\right]\times \pi \times d\times \Delta x\times \Delta t$$

The diffusion of water molecules may occur when there is a difference of moisture along the length of the pipe, but we ignore it assuming its effect is smaller than that of the Equations (A2) and (A3). We can take it into account if necessary by introducing a diffusion term

(A4) $$D\frac{\partial^{2}w}{\partial x^{2}}{\left(\frac{d}{2}\right)}^{2}\times \pi \times \Delta x\times \Delta t$$

The net increase of water molecules during a time interval Δt in the volume is

(A5) $$\frac{\partial w}{\partial t}{\left(\frac{d}{2}\right)}^{2}\times \pi \times \Delta x\times \Delta t$$

which is equal to a sum of Expressions (A2) and (A3),

(A6) $$\frac{\partial w}{\partial t}{\left(\frac{d}{2}\right)}^{2}\times \pi \times \Delta x\times \Delta t=-\frac{\partial w}{\partial x}v\cdot {\left(\frac{d}{2}\right)}^{2}\times \pi \times \Delta x\times \Delta t+\left[s\;k_{d}\;r-s\;k_{a}\;\left(1-r\right)\;w\right]\times \pi \times d\times \Delta x\times \Delta t$$

Then, the net increase of the adsorbed molecules on the inner surface during a time interval Δt is

(A7) $$s\frac{\partial r}{\partial t}\times \pi \times d\times \Delta x\times \Delta t$$

which is equal to the Expression (A3),

(A8) $$s\frac{\partial r}{\partial t}\times \pi \times d\times \Delta x\times \Delta t=\left[s\;k_{d}\;r-s\;k_{a}\;\left(1-r\right)\;w\right]\times \pi \times d\times \Delta x\times \Delta t$$

In a dimensionless form, the Equations (A6) and (A8) become

(A9) $$\frac{\partial r}{\partial \tau }=-a\times r+b\times \left(1-r\right)\times W$$

(A10) $$\frac{\partial W}{\partial \tau }+\frac{\partial W}{\partial \xi }=g\times a\times r-g\times b\times \left(1-r\right)\times W$$

where

(A11) $$\tau =\;\frac{t\times v}{L}$$

(A12) $$\xi =\;\frac{x}{L}$$

(A13) $$W=\;\frac{w\times L^{3}}{s\times L^{2}}$$

(A14) $$a=\;\frac{L\times k_{d}}{v}$$

(A15) $$b=\;\frac{s\times k_{a}}{v}$$

(A16) $$g=\;\frac{4\times L}{d}$$

In the equilibrium

(A17) $$\frac{\partial W}{\partial \tau }=\frac{\partial r}{\partial \tau }=0$$

therefore, from Equations (A9) and (A10),

(A18) $$W=constant=W_{0}$$

and

(A19) $$r=r_{1}=\frac{1}{1+\frac{a}{b}\times \frac{1}{W_{0}}}$$

This means that the adsorption ratio r reaches $r_{1}$ regardless of the position along the length of the pipe x when the gas with a constant moisture, $W=constant=W_{0}$, flows through the pipe for a long time. Furthermore, when a very dry gas comes in, no adsorption sites are occupied,

(A20) $$r_{1}\to 0\;\mathrm{when}\;\left(W_{0}\to 0\right)$$

whereas all site will be occupied when a very wet gas comes in.

(A21) $$r_{1}\to 1\;\mathrm{when}\;\left(W_{0}\to \infty \right)$$

which is obvious.

We can obtain the temporal evolution of the system by solving the Equations (A9) and (A10) under the initial condition,

(A22) $$W\left(\tau =0,\;\xi \right)=W_{0}\;\mathrm{for}\;\left(0\leq \xi \leq 1\right)$$

(A23) $$r\left(\tau =0,\;\xi \right)=r_{1}\;\mathrm{for}\;\left(0\leq \xi \leq 1\right)$$

and the boundary condition,

(A24) $$W\left(\tau ,\;\xi =0\right)=W_{1}\;\mathrm{for}\;(0<\tau )$$

We numerically solve the equations with the values of parameters taken from the experiments [14], but the coefficients $k_{d}$ and $k_{a}$ are arbitrary assumed such that a~1 and a~b. This is equivalent to the assumption that the contribution of adsorption and desorption is in the same order in the Equation (A9).
